# IFNγ signaling integrity in colorectal cancer immunity and immunotherapy

**DOI:** 10.1038/s41423-021-00735-3

**Published:** 2021-08-12

**Authors:** Wan Du, Timothy L. Frankel, Michael Green, Weiping Zou

**Affiliations:** 1 Department of Surgery, University of Michigan School of Medicine, Ann Arbor, MI USA; 2 Center of Excellence for Cancer Immunology and Immunotherapy, Rogel Cancer Center, University of Michigan School of Medicine, Ann Arbor, MI USA; 3 Department of Radiation Oncology, University of Michigan School of Medicine, Ann Arbor, MI USA; 4 Veterans Affairs Ann Arbor Healthcare System, University of Michigan School of Medicine, Ann Arbor, MI USA; 5 Graduate Programs in Immunology, University of Michigan School of Medicine, Ann Arbor, MI USA; 6 Tumor Biology, University of Michigan School of Medicine, Ann Arbor, MI USA; 7 Department of Pathology, University of Michigan School of Medicine, Ann Arbor, MI USA

**Keywords:** IFNGR, Interferon, MHC, Palmitoylation, T cell, EZH2, ARID1A, PD-1, PD-L1, Apoptosis, Ferroptosis, Colorectal cancer, Immunity, Oncology, Immunosuppression

## Abstract

The majority of colorectal cancer patients are not responsive to immune checkpoint blockade (ICB). The interferon gamma (IFNγ) signaling pathway drives spontaneous and ICB-induced antitumor immunity. In this review, we summarize recent advances in the epigenetic, genetic, and functional integrity of the IFNγ signaling pathway in the colorectal cancer microenvironment and its immunological relevance in the therapeutic efficacy of and resistance to ICB. Moreover, we discuss how to target IFNγ signaling to inform novel clinical trials to treat patients with colorectal cancer.

## Introduction

Early screening detection has improved the survival of patients with colorectal cancer. However, colorectal cancer remains one of the most common causes of cancer-related mortality in the USA and worldwide [1]. Immune checkpoint blockade (ICB) therapy is a new therapeutic approach to colorectal cancer. Based on the results of one phase III multicenter trial [2], the U.S. Food and Drug Administration (FDA) has approved Keytruda (pembrolizumab, anti-PD-1 monoclonal antibody) to treat a small subset of patients with colorectal cancer. Based on FDA approval, Keytruda can be used as the first-line treatment for patients with unresectable or metastatic microsatellite instability-high (MSI-H) or mismatch repair-deficient (dMMR) colorectal cancer without chemotherapy. This decision provides hope for patients with chemotherapy-resistant and late-stage MSI-H or dMMR colorectal cancer [3, 4]. Unfortunately, because few patients have these particular alterations, the vast majority of patients with colorectal cancer are not responsive to ICB therapy, highlighting the critical need to unveil cellular and molecular determinants of tumor resistance to immune-based therapies.

Tumor genetic and epigenetic alterations and immunosuppressive networks in the tumor microenvironment contribute to tumor resistance to ICB [5]. For example, β-catenin signaling [6], epigenetic regulation [7, 8], and other biological pathways [9–11] impair effector T-cell tumor trafficking and function. Loss-of-function mutations and genomic alterations in the IFN signaling pathway and antigen-presentation signaling pathways result in cancer immune evasion and support tumor resistance to ICB [12–15]. Notably, genetic mutations in the IFN signaling pathway and antigen-presenting machinery genes are infrequent in the majority of cancer patients, including colorectal cancer patients. Hence, it is essential to explore the immunotherapy resistance mechanisms in different types of human cancer, including colorectal cancer. IFN signaling, including type I IFNs (IFNα and IFNβ) and type II IFN (IFNγ), regulates tumor immune responses [16]. We focus on the IFNγ signaling pathway in this review. Recent studies have begun to dissect the mechanistic relationship between the integrity of the IFNγ signaling pathway and ICB resistance in the tumor microenvironment. Given the importance of the IFNγ signaling pathway in tumor immunity and immunotherapy, in this review, we summarize our current understanding of the IFNγ signaling pathway in colorectal cancer and discuss potential novel therapeutic approaches.

## Cellular sources of IFNγ in the colorectal cancer microenvironment

In the colorectal cancer microenvironment, tumor-infiltrating effector T cells and natural killer (NK) cells are the primary sources of IFNγ. Other minor contributors include Foxp3^+^CD4^+^ regulatory T cells (Tregs), Th17 cells, Th22 cells, NKT cells, innate lymphoid cells (ILCs), and antigen-presenting cells (APCs).

### CD8^+^ T cells

Tumor-infiltrating CD8^+^ T cells are among the most abundant producers of IFNγ and critically contribute to antitumor immunity [17–19]. Thus, a great deal of tumor-associated immunomodulatory strategies aim to alter CD8^+^ T-cell functions. In addition to the well-known immunosuppressive networks, including CD4^+^Foxp3^+^ Tregs, myeloid-derived suppressor cells (MDSCs), and immune inhibitory macrophages [5], recent studies have demonstrated novel mechanisms affecting CD8^+^ T-cell function, including altering IFNγ expression in the colon cancer microenvironment. For example, during sporadic intestinal tumorigenesis, mitophagy in colon intestinal epithelial cells causes lysosomal membrane permeabilization through iron accumulation, subsequently enhancing IFNγ expression in CD8^+^ T cells and augmenting major histocompatibility complex-class I (MHC-I) presentation in dendritic cells (DCs) [20]. However, cancer cell-associated mechanisms often inhibit IFNγ production by suppressing CD8^+^ T-cell tumor trafficking, survival, and function. For example, tumor cells highly express the methionine transporter SLC43A2, which can compete for methionine metabolism in CD8^+^ T cells, leading to decreased activation of STAT5 in CD8^+^ T cells and subsequent impairment of CD8^+^ T-cell IFNγ production in tumor-bearing mice and patients with colorectal cancer [21]. In addition, cholesterol can reduce IFNγ production in CD8^+^ T cells in colon cancer by increasing endoplasmic reticulum (ER) stress [22]. Inhibiting the ER stress sensor X-box binding protein 1 reduces cholesterol in CD8^+^ T cells and can restore antitumor activity. The intestinal microbiota may also impact CD8^+^ T-cell IFNγ production. Some bacterial strains from healthy human donor feces can promote IFNγ^+^CD8^+^ T cells in the intestine and enhance the efficacy of ICB in colon cancer-bearing mice [23]. Thus, multiple layers of regulatory mechanisms can affect IFNγ production by CD8^+^ T cells in the colon cancer microenvironment.

### CD4^+^ T helper (Th) subsets

While Th1 cells can be an important source of IFNγ, these cells can be functionally altered in the tumor microenvironment [24]. Metabolism and particularly aerobic glycolysis regulate CD4^+^ T-cell function and IFNγ production. CD4^+^ T cells cultured with galactose, a monosaccharide that can enter glycolysis, manifest severe defects in IFNγ production [25]. Deficiency in lactate dehydrogenase A, an essential enzyme in glycolysis, leads to diminished IFNγ expression in CD4^+^ T cells under Th1 conditions [26]. Th1 cell IFNγ production is also regulated by signaling factors and immunosuppressive immune cells in the cancer microenvironment. For example, TGFβ [27], transcription factor p73 (tumor protein p73) [28], Tregs [29], and MDSCs [30] can inhibit the expression of IFNγ in Th1 cells.

Aside from Th1 cells, other human colon cancer-infiltrating CD4^+^ T-cell subsets, including Th17 cells [31, 32], Th22 cells, and Tregs, can express IFNγ. The role of Th17 cells in colorectal cancer is controversial, with some studies suggesting a protumorigenic function and others demonstrating enhanced tumor immunity [32]. Th22 cells promote colorectal cancer cell stemness and cancer progression through an IL-22–STAT3-dependent pathway in the colorectal cancer microenvironment [33]. However, the role of IFNγ produced by Th17 cells and Th22 cells has not been specifically studied in this or other types of human cancer. Despite the fact that Tregs suppress the cancer immune response via multiple pathways [34, 35], Tregs also express IFNγ, and IFNγ^+^ Tregs remain immunologically suppressive in the human colorectal cancer microenvironment [36, 37]. Neuropilin‐1 is required for the stability and function of tumor-infiltrating Tregs. The loss of neuropilin‐1 alters the Treg phenotype and facilitates tumor elimination [38]. In addition, ablation of the nuclear factor κB subunit c-Rel increases IFNγ expression in Tregs, thereby delaying tumor growth [39]. Disruption of the CARMA1-BCL10-MALT1 signalosome complex in mature Tregs enhances the production of IFNγ in the tumor microenvironment, resulting in stunted tumor growth [40].

Hence, different T-cell subsets can express IFNγ, thereby altering the immune responses in the colorectal cancer microenvironment.

### NK cells

NK cells are another major source of IFNγ during immune responses [41]. NK cells rapidly produce IFNγ upon activation and exert antitumor functions. However, tumor progression may lead to NK cell exhaustion, thereby limiting the antitumor potential of NK cells. Blockade of the checkpoint receptor TIGIT (T-cell immunoglobulin and immunoreceptor tyrosine-based inhibitory motif domain) can reverse the exhaustion of tumor-infiltrating NK cells and promote IFNγ production in a colon cancer-bearing mouse model [42].

### NKT cells

NKT cells have the potential to produce both proinflammatory and anti-inflammatory cytokines [43]. This differential cytokine production depends on the environment at the time of NKT cell activation. Stimulation via IL-12 receptor or NKR-P1 (a prototypical NK cell receptor) preferentially induces NKT cell IFNγ production [44], which is vital for antitumor activity [45]. Similarly, basic helix–loop–helix transcription factor family member e40 (Bhlhe40) is highly expressed in NKT cells and functions as a cofactor for T-box transcription factor Tbx21 (T-bet), enhancing IFNγ production in NKT cells. Experimental evidence suggests that Bhlhe40-deficient NKT cells have impaired IFNγ production and diminished antitumor effects [46].

### ILCs

Based on the expression of master transcription factors and effector cytokines, ILCs are classically divided into three major groups: ILC1s, ILC2s, and ILC3s. ILC1s depend on T-bet for their development, can produce IFNγ, and may function in tumor immune surveillance and clearance [47]. In the late stage of colorectal cancer, ILC1s are decreased and produce less IFNγ [48]. However, ILCs are functionally plastic, and their capacity for IFNγ production can be regulated [49]. For example, the transcription factor GATA-binding protein 3 (GATA3) in ILC2s binds to the regulatory element of ILC effector genes, thereby restricting IFNγ production [50]. ILC3s can produce high levels of IFNγ and exhibit some degree of plasticity, as IL-12 can drive the conversion of these cells to IFNγ-producing ILC1s [51].

### APCs

IL-12 and IL-18 can stimulate APCs, including DCs and macrophages, to produce IFNγ [52, 53]. Human melanomas harbor IFNγ-producing macrophages in the tumor microenvironment [54]. The biological significance of APC-derived IFNγ has not been defined in colon cancer immunity.

In summary, IFNγ can be expressed by multiple immune cell subsets in the colorectal cancer microenvironment. The relative contribution of each cell type to the total levels of IFNγ may depend on the quantity and quality of each immune subset within the tumor and is likely subject to multiple layers of regulation in the colorectal cancer microenvironment.

## IFNγ signaling regulation in the colorectal cancer microenvironment

**Fig. 1:**
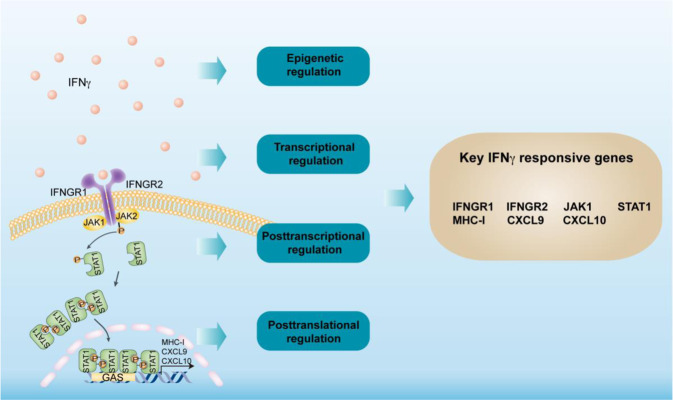
IFNγ signaling pathway genes. IFNγ binds to the IFNγ receptor (IFNGR) complex, recruiting JAK1 and JAK2 to the receptor complex and inducing the subsequent phosphorylation of STAT1. Then, phosphorylated STAT1 dimers translocate to the nucleus and induce the transcription of ISGs (interferon-stimulated genes) by binding GAS (IFN gamma-activated sequences) in the gene promotors. The IFNγ signaling pathway can be regulated at the epigenetic, transcriptional, posttranscriptional, and posttranslational levels

The IFNγ signaling pathway is a well-controlled molecular network. IFNγ binds to IFNγ receptors (IFNGRs) and stimulates the Janus kinase (JAK)–signal transducer and activator of transcription (STAT) signaling pathway, which in turn activates an IFN-stimulated gene (ISG) transcriptional program and regulates the immune response. The suppressor of cytokine signaling (SOCS) protein family (primarily SOCS1 and SOCS3) is a well-known negative regulator of the IFNγ signaling pathway [55]. Hence, we focused on the regulation of the IFNγ signaling pathway at the epigenetic, transcriptional, posttranscriptional, and posttranslational levels in the context of cancer immunity (Fig. 1).

### Epigenetic regulation

Epigenetic histone modifications by polycomb repressive complex 2 (PRC2) and SWItch/Sucrose Non-Fermentable (SWI/SNF) complexes are involved in the regulation of the IFNγ signaling pathway in colorectal cancer. This regulation occurs in part through the control of Th1-type chemokines, such as chemokine (C-X-C motif) ligand 9 (CXCL9) and CXCL10, which regulate effector T-cell recruitment to the colorectal cancer microenvironment. Enhancer of zeste homolog 2 (EZH2), a PRC2 component, mediates histone H3 lysine 27 trimethylation and represses tumor production of CXCL9 and CXCL10 [7, 56]. Conversely, ARID1A (BAF250A), a core member of the SWI/SNF complex, supports CXCL9 and CXCL10 expression in human colorectal cancer cells, resulting in enhanced recruitment of IFNγ-producing immune cells [57]. It has been reported that genetic deficiency in ARID1A results in a reduction in chromatin accessibility at Th1-type chemokine loci in tumor cells, including colon cancer cells, and ARID1A interacts with EZH2 via its carboxyl terminal, thereby restraining the inhibitory effect of EZH2 on IFNγ signaling-mediated gene expression [57]. In addition, EZH2 can regulate IFNγ signaling by silencing endogenous retroviruses (ERVs). A subclass of ERVs, termed stimulated 3 prime antisense retroviral coding sequences (SPARCS), undergo positive feedback signal amplification due to antisense localization in the 3ʹ-untranslated region (3ʹ-UTR) of ISGs. EZH2 can silence the effect of SPARCS in H69AR human small cell lung cancer cells [58]. Furthermore, as an additional mode of epigenetic modification, histone deacetylase (HDAC) and histone acetyltransferase dynamically regulate STAT1 acetylation, which counteracts IFN-induced STAT1 phosphorylation, nuclear translocation, DNA binding, and target gene expression. The phospho-acetyl switch regulates STAT1 signaling via CREB binding protein, HDAC3, and T-cell protein tyrosine phosphatase (TCP45). HDAC inhibitors block IFNγ-induced STAT1 phosphorylation of a critical tyrosine residue in the STAT1 C-terminus in hematopoietic cells [59–61]. It remains to be determined whether these types of epigenetic regulation occur in colon cancer cells.

**Fig. 2:**
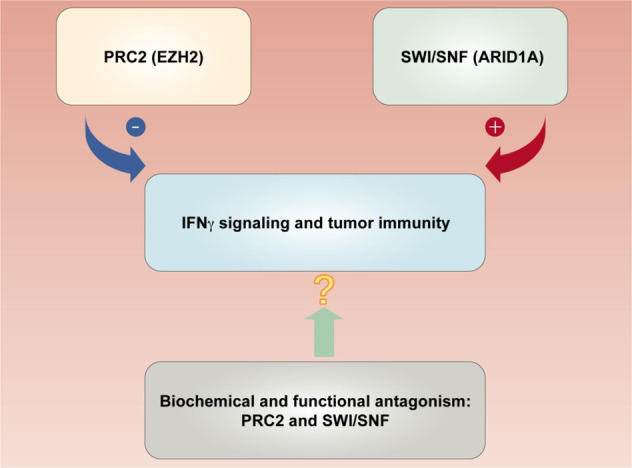
Cross-talk between PRC2 and SWI/SNF complexes in regulating the IFNγ signaling pathway and tumor immunity. The PRC2 component EZH2 represses IFNγ signaling genes (CXCL9 and CXCL10), diminishing effector T-cell tumor trafficking. The SWI/SNF complex core member ARID1A antagonizes EZH2 and enhances IFNγ signaling gene expression, promoting T-cell tumor migration and antitumor immunity. Targeting PRC2 and SWI/SNF complexes may potentiate ICB therapy

DNA methylation by DNA methyltransferases (DNMTs) and demethylation by ten–eleven translocation family of protein 2 (TET2) can also regulate the IFNγ signaling pathway in tumor cells. DNMT1 suppresses tumor production of CXCL9 and CXCL10 and subsequently reduces T-cell tumor migration [7]. In addition, IFNγ stimulation results in the phosphorylation and nuclear translocation of STAT1, leading to STAT1–TET2 associations. Many IFNγ-responsive genes, including PD-L1, CXCL9, CXCL10, and CXCL11, are silenced via DNA methylation. TET2-mediated DNA demethylation increases 5hmC levels on the promoters of these IFNγ-responsive genes, thereby promoting antitumor immunity [62]. Thus, epigenetic regulation of the IFNγ signaling pathway can affect tumor immunity and immunotherapy (Fig. 2).

### Transcriptional regulation

Repetitive elements (REs) maintain genomic stability and drive human genome diversity. F-Box protein 44 (FBXO44) has been identified as an essential repressor of REs in a panel of cancer cells, including colon cancer cell lines. FBXO44 recruits SUV39H1 to REs, which is essential for H3K9me3-mediated transcriptional silencing of REs in cancer cells. FBXO44 inhibition reactivates REs, leading to IFNγ signaling activation in cancer cells, as shown by the increased expression of IFNGR1, IFNGR2, and other ISGs and the decreased expression of protein tyrosine phosphatase nonreceptor type 2 (PTPN2), an IFNγ signaling inhibitor [9]. Therefore, FBXO44/SUV39H1 inhibition can enhance cancer cell immunogenicity and overcome ICB resistance via the transcriptional regulation of IFNγ signaling [63].

Moreover, phosphatidylinositol 3-kinase (PI3K) can mediate transcriptional regulation of the IFNγ signaling pathway in tumors. There is reciprocal regulation between the IFNγ signaling pathway and PI3K. While IFNγ signaling activates PI3K, PI3K simultaneously transcriptionally and translationally induces IFN-responsive gene expression in mouse embryonic fibroblasts [64]. In addition, long noncoding RNAs (lncRNAs) may be involved in the regulation of IFNγ target gene expression. For example, the lncRNA LIMIT can cis-activate the guanylate binding protein gene cluster, disrupting the association between heat shock protein 90 and heat shock factor-1 (HSF1). This disruption results in HSF1 activation and the transcriptional upregulation of MHC-I in several types of cancer cells, including colon cancer cells [65]. Thus, the IFNγ signaling pathway can be modulated at the transcriptional level via multiple distinct mechanisms.

### Posttranscriptional regulation

Several posttranscriptional mechanisms have been reported to modulate IFNγ production in T cells, including tumor-infiltrating T cells. CD28 costimulation [66] and protein kinase C activation [67] contribute to IFNγ mRNA stabilization and IFNγ protein production in T cells. Similarly, a lack of adenylate-uridylate-rich elements (AREs) within the 3ʹ-UTR maintains IFNγ mRNA stability and enhances IFNγ protein expression in tumor-infiltrating T cells [66]; impaired aerobic glycolysis, which frequently occurs in the tumor microenvironment, leads to enhanced GAPDH binding to IFNγ AREs, thereby reducing IFNγ expression [25]. Adenosine-to-inosine editing in double-stranded RNA is a highly prevalent posttranscriptional modification, and this modification is catalyzed by adenosine deaminase acting on RNA (ADAR) enzymes. The absence of ADAR1 editing results in the upregulation of IFNγ-responsive gene expression [68] and increases double-stranded RNA ligand sensing and IFN signaling in tumors [68]. Consistent with this, tumor ADAR1 deficiency sensitizes CT26 and MC38 mouse colon cancers to ICB in mouse models [11].

### Posttranslational modification

Posttranslational modifications of IFNγ signaling mediators, such as IFNGR and JAK/STAT1, through palmitoylation, phosphorylation, and SUMOylation are critical regulators of IFNγ signaling. IFNGRs, including IFNGR1 and IFNGR2, are essential elements in the IFNγ signaling pathway. IFNGR1 in colorectal cancer cells can be palmitoylated, which allows its interaction with AP3D1, a lysosome sorting adapter, and facilitates IFNGR1 lysosomal sorting and degradation. Thus, IFNGR1 palmitoylation promotes IFNGR1 degradation and instability in colorectal cancer cells [69]. IFNGR1 also undergoes rapid K48 polyubiquitination, which is modulated by glycogen synthase kinase 3 beta (GSK3β), in epithelial cells and monocytic cell lines. GSK3β inhibition can destabilize IFNGR1 [70]. Bruton tyrosine kinase-mediated phosphorylation of IFNGR2 at tyrosine 289 promotes IFNGR2 membrane translocation in HEK293T cells [71]. This translocation is required for IFNGR2 to form a functional heterodimer with IFNGR1 to sense extracellular IFNγ. However, it remains to be determined whether this regulation of IFNGR2 occurs in colorectal cancer cells.

JAK1 and STAT1 mediate IFNGR signal transduction. PTPN2 dephosphorylates JAK1 and STAT1 and negatively regulates IFNγ signaling. The loss of PTPN2 results in an increase in tumor antigen presentation and T-cell trafficking due to enhanced expression of IFNγ-responsive genes, including MHC-I, Cxcl9, Cxcl10, Cxcl11, and Ccl5 [9]. JAK1 and IFNGR1 can also be modified by the scaffold protein Ajuba LIM protein (AJUBA). AJUBA specifically binds the FERM domain (F for 4.1 protein, E for ezrin, R for radixin, and M for moesin) of JAK1 and blocks the interaction between JAK1 and IFNGR1. Consequently, AJUBA suppresses IFNγ-stimulated STAT1 phosphorylation and translocation, promoting colorectal cancer growth [72].

Small ubiquitin-like modifier (SUMO) overexpression leads to STAT1 SUMOylation, thereby reducing IFNγ-induced STAT1 phosphorylation. The IFNγ transcriptional response is sensitive to SUMO, and ginkgolic acid mediates the inhibition of SUMOylation, resulting in high IFNγ-induced STAT1 phosphorylation in HeLa cells [73]. Thus, the IFNγ signaling pathway is subject to a wide variety of regulatory posttranslational modifications and could be a target for the modulation of antitumor immunity.

## Genetic mutations and the loss of IFNγ signaling genes

Mutations in the components of the IFNγ signaling pathway have been reported in multiple types of human cancer, including colorectal cancer (Table 1). Notably, the loss of IFNGR expression has been identified in colorectal cancer [69].

**Table 1:** Mutations in the IFNγ signaling pathway in colorectal cancer

Mutant gene	Mutation types	References
JAK1	Frameshift	[76]
	Homozygous Q503* nonsense loss-of-function mutation	[12]
	Homozygous W690* nonsense loss-of-function mutation	[14]
JAK2	F547 splice-site mutation	[12]
B2M	Loss of heterozygosity	[76]

### JAK mutations

Tumors with a high mutational burden are more likely to respond to ICB therapy. However, some patients fail to respond despite having a high mutational load. Inactivating JAK1/JAK2 mutations are detected in some tumor types (particularly melanoma), making these mutations candidates for observed ICB resistance. Whole-exome sequencing has revealed homozygous loss-of-function mutations with a Q503* nonsense mutation in the gene encoding JAK1, an F547 splice-site mutation in the gene encoding JAK2, and a 4-bp S14 frameshift deletion in exon 1 of the beta-2-microglobulin component of MHC-class I in patients with metastatic melanoma who are resistant to ICB therapy [12]. *JAK1*-mutated cells fail to upregulate ISGs, such as JAK2, STAT1, STAT3, IRF1, PD-L1, and PD-L2, following IFNγ stimulation. *JAK2*-mutated cells present a complete loss of IFNγ-induced JAK-STAT genes, such as IRF1 and PD-L1 [74]. Truncating mutations, homozygous deletions, and low protein levels of IFNGR1, IFNGR2, JAK1, JAK2, STAT1, and IRF1 in melanoma patients result in shorter survival than that of patients with wild-type IFNγ signaling genes [75]. Furthermore, patients with loss-of-function mutations in JAK1/2 fail to respond to ICB therapy [14]. Thus, JAK1 and JAK2 mutations may contribute to ICB resistance in patients with these genetic mutations [12]. However, genetic mutations in IFN signaling genes are infrequent in colorectal cancer patients, occurring in less than 10% of patients with colorectal adenocarcinoma [14]. Loss-of-function alterations, including JAK1 frameshifts, are found in less than 3% of microsatellite instability-low (MSI-L) colon adenocarcinoma samples [76], which make up 85% of colorectal cancer patients [77]. Given that a vast majority of colorectal cancer patients do not have mutations in IFNγ signaling genes, it is unlikely that this represents a major contribution to ICB resistance in colorectal cancer patients.

### MHC-I complex mutations

The MHC-I complex consists of an HLA gene encoding heavy chains and a B2M gene encoding a light chain. B2M mutations are found in 3.4% of patients with colorectal cancer [78]. B2M aberrations contribute ICB resistance in patients with colorectal cancer [13].

### Loss of optineurin and IFNGR1 expression

**Fig. 3:**
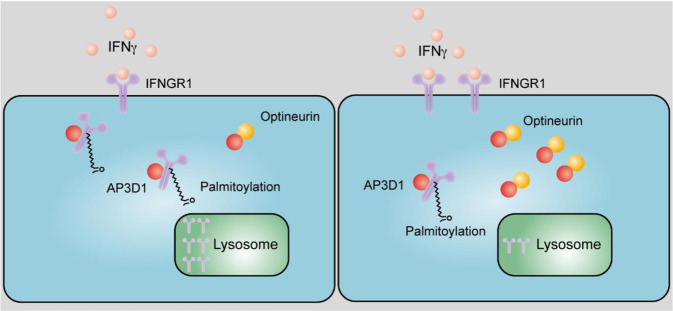
Lysosomal sorting and degradation of palmitoylated IFNGR1. AP3D1 recognizes the palmitoylation signal and then binds and sorts palmitoylated IFNGR1 to lysosomes for degradation. Optineurin competes with AP3D1 for IFNGR1 binding and prevents IFNGR1 lysosomal sorting and degradation. The loss of optineurin impairs the integrity of the IFNγ signaling pathway in colorectal cancer. Low (left) and high (right) optineurin expression

Given that colorectal cancer patients exhibit infrequent IFN- and MHC-signaling gene mutations and are generally resistant to ICB, a recent study has explored alternative mechanisms that may constrain IFNγ signaling in colorectal cancer [69]. This report demonstrates that optineurin is a shared node between the IFN- and MHC-signaling gene pathways, and the loss of optineurin occurs in early stage human colorectal cancer. Interestingly, optineurin deficiency accelerates IFNGR1 degradation and abolishes MHC-I expression. This deficiency impairs T-cell-mediated immunity and diminishes immunotherapy efficacy in murine cancer models and cancer patients. Thus, loss of optineurin impairs the integrity of the IFNγ- and MHC-I-signaling pathways via IFNGR1 degradation, thereby driving immune evasion and intrinsic immunotherapy resistance in colorectal cancer [69] (Fig. 3). Thus, while it is evident that JAK1, JAK2, and B2M mutations can contribute to immune resistance in multiple types of cancer, the loss of IFNγ signaling gene expression may be the predominant source of ICB resistance in colorectal cancer.

## Dual effects of IFNγ

**Fig. 4:**
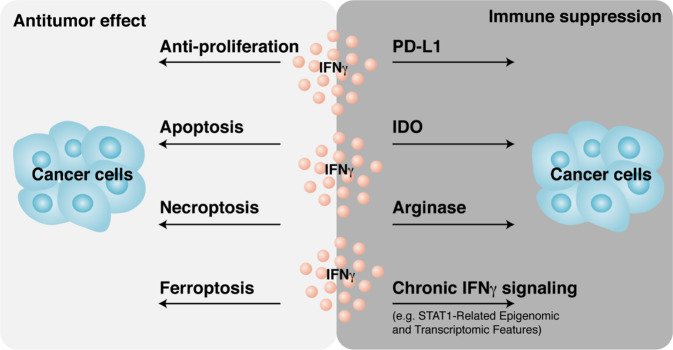
Dual effects of IFNγ in cancer immunity. IFNγ exerts antitumor effects via multiple mechanisms. In contrast, IFNγ can protect cancer cells from immune cell attack by inducing PD-L1, IDO, and arginase expression. Chronic IFNγ signaling enforces immunosuppressive mechanisms in the tumor microenvironment

IFNγ gene signaling promotes spontaneous and therapy-induced anticancer immunity. However, accumulating evidence suggests dual effects wherein IFNγ signaling promotes cancer development and immune evasion (Fig. 4).

### Role of IFNγ in antitumor effects

**Fig. 5:**
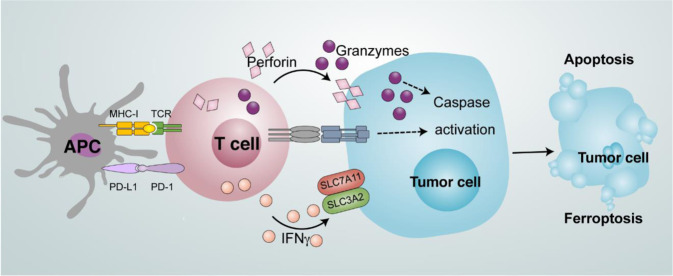
Mode of tumor cell death induced by T cells. Antigen-presenting cells (APCs) prime and activate T cells. T cells can induce tumor cell apoptosis by secreting perforin and granzymes. In addition, T cells promote tumor cell ferroptosis via IFNγ-mediated downregulation of SLC3A2 and SLC7A11

IFNγ signaling plays a critical role in antitumor immunity. IFNγ stimulates the expression of MHC-I and MHC-II in tumor cells and APCs, enhances IL-12 production by APCs, facilitates Th1 polarization, and promotes T-cell and NK cell tumor trafficking via Th1-type chemokine production in the tumor microenvironment. Moreover, IFNγ can exert a direct anticancer effect on cell proliferation [79] and induce cancer cell apoptosis [80] and necroptosis [81]. Furthermore, IFNγ downregulates the expression of SLC3A2 and SLC7A11, two subunits of the glutamate–cystine antiporter system xc−, impairs the uptake of cystine by tumor cells, and subsequently promotes tumor cell lipid peroxidation and ferroptosis [82, 83] (Fig. 5). Notably, IFNγ is one of the players that induces tumor cell death, including apoptosis, necroptosis, and ferroptosis. The nature of IFNγ-regulated tumor cell death may depend on specific underlying mechanisms, the partners of IFNγ, and tumor cell type in the tumor microenvironment [83].

Given that IFNγ is often released by activated CD8^+^ T cells, recent studies have examined how far IFNγ can reach within the tumor microenvironment. These studies showed that IFNγ sensing can occur at long distances from antigen-positive (Ag)^+^ zones into Ag^−^ zones, indicating a bystander effect of IFNγ [84, 85]. These reports suggest that the spatiotemporal regulation of IFNγ signaling is important in antitumor immune responses, including tumor-associated antigen-specific and bystander effects, and regulates tumor cell death (apoptosis, necrosis, and ferroptosis).

### Role of IFNγ in cancer immune evasion

In addition to antitumor effects, IFNγ can contribute to tumor immune evasion. For example, IFNγ induces the expression of immune inhibitory molecules, including B7-H1 (PD-L1), indoleamine 2,3-dioxygenase (IDO), and arginase, in the tumor microenvironment. PD-L1 is expressed on tumor cells and immune cells, particularly APCs in tumor-draining lymph nodes and the tumor microenvironment [86–89]. IFNγ strongly stimulates PD-L1 expression in the tumor microenvironment, thereby hampering antitumor immunity and ICB therapy [86, 90]. IDO is a kynurenine pathway enzyme that catalyzes the first and rate-limiting step in tryptophan catabolism to form N-formyl-kynurenine. IDO is expressed in tumor cells, fibroblasts, and immune cells infiltrating the tumor microenvironment. IDO contributes to an immune-tolerant tumor microenvironment and correlates with poor prognosis in a wide spectrum of cancer types, including colorectal cancer. IFNγ is a potent inducer of IDO expression, which serves as a pathogenic driver of colorectal cancer progression. Kynurenine metabolites activate PI3K-Akt signaling in the neoplastic epithelium, promoting cellular proliferation and resistance to apoptosis. Intestinal epithelium-specific IDO knockout results in decreased colon tumorigenesis in a mouse model of colon cancer [91]. However, inhibition of IDO1 as an anticancer approach remains uncertain. A phase III, randomized, double-blind clinical study using the IDO1 selective inhibitor epacadostat in combination with pembrolizumab failed to improve progression-free survival or overall survival compared with pembrolizumab monotherapy in patients with unresectable or metastatic melanoma [92]. The role of IFNγ in IDO1 production in this trial remains to be determined. Arginase is an enzyme that hydrolyzes arginine to ornithine and urea. IFNγ induces arginase expression in many different types of cells [93, 94]. Arginase contributes to the immunosuppressive activities of macrophages, DCs, and MDSCs in the tumor microenvironment by metabolizing nutrients that are key to CD8^+^ T-cell activation [94–96].

In summary, the dynamic and kinetic impact of IFNγ on immunogenicity and immune evasion may determine the fate of tumor progression. In line with this notion, exposure to persistent IFNγ signaling allows tumors to acquire immune resistance and augments the expression of immune inhibitory molecules [97]. Hence, the immunogenic action of IFNγ may be inevitably accompanied by an elevated immune evasion mechanism (PD-L1, IDO1, and Arg1) in the tumor microenvironment, and a specific therapeutic combination may overcome this unwanted effect. Based on this finding, a variety of combinatory approaches with ICB are being explored (Table 2) [98]. Blocking the aryl hydrocarbon receptor pathway in IDO-expressing tumors would overcome the limitation of single IDO-targeting agents and improve the efficacy of combination therapy with ICB [99]. A selective ARG1/2 inhibitor (OATD-02) has shown antitumor activities in preclinical tumor models alone or in combination with anti-PD-1 [100]. Thus, targeting IFNγ-induced intrinsic immunosuppressive mechanisms should be explored in patients with colorectal cancer.

**Table 2:** Ongoing clinical trials of checkpoint inhibitor combination therapy in colorectal cancer

Combined agents	Combination	Treatment setting	Study population	Phase	Trial identifier
Checkpoint inhibitor	aPD-1 + aCTLA-4	Nivolumab + ipilimumab	Metastatic colorectal cancer MSI-H colorectal cancer	Phase II	NCT04730544
Chemotherapy	Chemotherapy + aPD-1	Pemetrexed + oxaliplatin + dexamethasone + pembrolizumab + dietary supplement (folic acid + vitamin B-12)	Metastatic colorectal cancer	Phase I	NCT03626922
	Chemotherapy + aPD-L1	5-FU + avelumab	MSI-H colon cancer	Phase III	NCT03827044
	Chemoradiation + aPD-L1	5-FU + capecitabine Pill + radiotherapy + avelumab + surgical resection	Locally advanced rectal cancer	Phase II	NCT03299660
Radiotherapy	Stereotactic ablative radiotherapy + aPD-L1	SABR + atezolizumab	Colorectal cancer	Phase II	NCT02992912
	Radiotherapy + aPD-1	Radiotherapy + radiofrequency ablation + pembrolizumab	Metastatic colorectal cancer	Phase II	NCT02437071
	Radiotherapy + chemotherapy + aPD-1	Radiotherapy + capecitabine + durvalumab	Rectal cancer	Phase II	NCT04083365
Target therapy	VEGFR inhibitor + aPD-L1	Cabozantinib + durvalumab	Colorectal cancer	Phase I/II	NCT03539822
	VEGF inhibitor + aPD-L1 + PARP inhibitor	Cediranib + MEDI4736 + olaparib	Colorectal cancer	Phase I/II	NCT02484404
	EGFR inhibitor + aPD-L1 + chemotherapy	Cetuximab + avelumab + irinotecan	MSS refractory metastatic colorectal cancer	Phase II	NCT03608046
DNMT inhibitor	DNMT inhibitor + aPD-1	Azacytidine +pembrolizumab	Metastatic colorectal cancer	Phase II	NCT02260440
Cancer vaccine	Cancer vaccine + aPD-1	Galinpepimut-S + pembrolizumab	Colorectal cancer	Phase I/II	NCT03761914
Monoclonal microbial	Monoclonal microbial + aPD-1	EDP1503 vs EDP1503 + pembrolizumab	Metastatic colorectal cancer	Phase I/II	NCT03775850

## The IFNγ signaling pathway and colorectal cancer immunotherapy

### ICB in colorectal cancer

The FDA has approved two PD-L1/PD-1 signaling-blocking antibodies, pembrolizumab and nivolumab, for the treatment of patients with MSI-H or dMMR metastatic colorectal cancer. As ~15% of colorectal cancer patients exhibit MSI-H or dMMR [101–103], the vast majority of colorectal cancer patients do not benefit from ICB. Several ongoing clinical trials are evaluating the efficacy of ICB in combination with chemotherapy, radiotherapy, and target therapies in colorectal cancer patients (Table 2). Combinations of multiple immune-based therapies, such as CTLA-4 and PD-1 blockers, have yielded improved progression-free survival and overall survival rates in patients with dMMR-MSI-H metastatic colorectal cancer [104, 105]. As chemotherapy has pleiotropic immunomodulatory effects [106, 107], immunogenic chemotherapy could sensitize tumors to ICB [108]. FOLFOX is the primary chemotherapy regimen for the treatment of colorectal cancer and includes folinic (FOL), fluorouracil (F), and oxaliplatin (OX). The combination of FOLFOX and anti-PD-1 improves tumor control in colorectal cancer-bearing mice [109]. However, the efficacy of this combination in patients has not yet been established [110, 111]. In metastatic cancers, radiotherapy is a powerful adjuvant for immunotherapy, occasionally amplifying clinical efficacy and improving patient survival [112]. The combination of radiation therapy and ICB is well tolerated in patients [113]. However, the efficacy of this combination is limited in MSS colorectal cancer patients [114]. Targeted therapies can impede tumor growth and induce immune attack. The vascular endothelial growth factor receptor (VEGFR) signaling pathway can mediate T-cell inhibition and increase the tumor recruitment of Tregs and MDSCs [115]. The combination of VEGF/VEGFR inhibitors and ICB may generate clinical benefits for colorectal cancer patients. It appears that this combination has a manageable safety profile. However, the objective tumor response rate remains limited in MSS colorectal cancer patients [116, 117].

Additional clinical trials are exploring other combinations. Cancer vaccines may trigger cytotoxic antitumor immune responses to multiple tumor-specific antigens, including neoantigens [118]. Current clinical trials are testing the combination of cancer vaccines and ICB in colorectal cancer patients (Table 2). The interplay between commensal bacteria and immune cells can affect systemic and local immunity in the gut [119]. The combination of the monoclonal antimicrobial EDP1503 with ICB may enhance the antitumor response in metastatic colorectal cancer patients. This combination is currently in phase I/II studies (Table 2).

Given that most of these clinical trials are in phase I/II, the therapeutic efficacy has yet to be determined. How to target colorectal cancer patients with MSI-L, MSS, or proficient mismatch repair remains a significant challenge scientifically and clinically.

### Targeting the IFNγ signaling pathway in colorectal cancer therapy

**Fig. 6:**
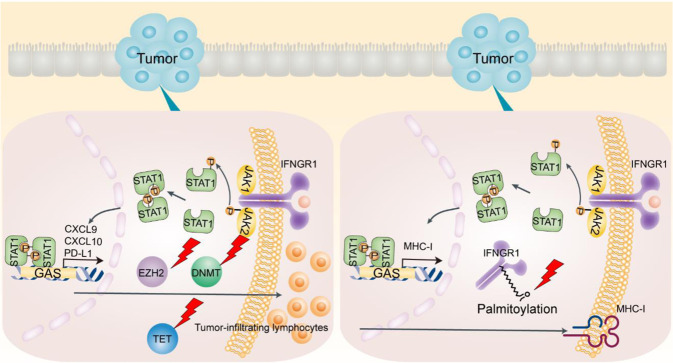
Targeting the IFNγ signaling pathway in colorectal cancer therapy. Targeting EZH2 (enhancer of zeste homolog 2), DNMTs (DNA methyltransferases), TET (ten–eleven translocation family of protein 2), and palmitoylation can rescue and stimulate the expression of key IFNγ signaling genes and enhance antigen presentation, T-cell activation, and T-cell trafficking in colorectal cancer

The loss of IFNγ signaling gene expression has been observed in patients with colorectal cancer. Strategies that enhance IFNγ signaling are a rational and novel approach for the management of colorectal cancer patients (Fig. 6).

As epigenetic silencing decreases Th1-type chemokines to limit effector T-cell trafficking to the tumor, ICB in combination with inhibitors of EZH2 and DNMT1 slows cancer progression in ID8 ovarian cancer [7] and CT26 colon models [120]. Clinical trials with the combination of DNMT inhibitors and ICB are in the early stages [121]. One phase II study showed that pembrolizumab (anti-PD-1 antibody) plus azacytidine (DNMT inhibitor) was feasible with a tolerable safety profile. However, this combination yielded minimal antitumor effects for MSS metastatic colorectal cancer [121]. It remains to be determined whether azacytidine affects the IFNγ signaling pathway in these patients and whether other DNMT inhibitors can be evaluated clinically.

The loss of TET2 diminishes IFN signaling and impairs Th1-type chemokine expression in murine colon cancer MC38 cells. Vitamin C/l-ascorbic acid can stimulate TET activity, thereby enhancing Th1-type chemokine expression and T-cell tumor infiltration and leads to enhanced antitumor immunity and ICB efficacy in mice with transplanted B16-OVA cells [62]. Thus, vitamin C could potentially be used in conjunction with ICB to enhance efficacy.

Given that IFNGR1 palmitoylation is essential for its interaction with AP3D1 and subsequent IFNGR1 lysosomal sorting and degradation in colon cancer, suppression of IFNGR1 palmitoylation can restore cancer IFNγ signaling integrity and sensitize colorectal cancer cells to immunotherapy [69]. Targeting IFNGR1 stability, including palmitoylation, may be a promising approach to overcome intrinsic ICB resistance in patients with colorectal cancer.

## Conclusion

ICB has been approved to treat colorectal cancer patients with dMMR-MSI-H metastatic disease. However, not all patients with dMMR-MSI-H and virtually none without these alterations effectively respond to ICB. To improve the outcomes of colorectal cancer patients, combinatorial therapies with ICB are being explored in different clinical trials. Most of these early clinical trials show acceptable safety profiles. Given the importance of the IFNγ signaling pathway in colorectal cancer immunity and that dysfunctional IFNγ signaling in tumor cells is a mechanism of immunotherapy resistance, it is critical to study the kinetic changes in the IFNγ signaling pathway during the course of ICB in patients with colorectal cancer. New clinical applications stem from scientific breakthroughs via basic research and discovery, and a deeper understanding of IFNγ signaling pathway integrity in colorectal cancer microenvironments is critical. New insights into the genetic, epigenetic, and metabolic regulation of IFNγ signaling will pave the way for new clinical trials and novel immune-based therapies for patients with colorectal cancer.
